# Automated extraction of pod phenotype data from micro-computed tomography

**DOI:** 10.3389/fpls.2023.1120182

**Published:** 2023-02-24

**Authors:** Evangeline Corcoran, Laura Siles, Smita Kurup, Sebastian Ahnert

**Affiliations:** ^1^ Environment and Sustainability Theme, AI for Science and Government Programme, The Alan Turing Institute, London, United Kingdom; ^2^ Department of Plant Sciences for the Bioeconomy, Rothamsted Research, Harpenden, United Kingdom; ^3^ Department of Chemical Engineering and Biotechnology, School of Technology, University of Cambridge, Cambridge, United Kingdom

**Keywords:** phenotyping, plant development, machine learning, computer vision, micro-compute tomography

## Abstract

**Introduction:**

Plant image datasets have the potential to greatly improve our understanding of the phenotypic response of plants to environmental and genetic factors. However, manual data extraction from such datasets are known to be time-consuming and resource intensive. Therefore, the development of efficient and reliable machine learning methods for extracting phenotype data from plant imagery is crucial.

**Methods:**

In this paper, a current gold standard computed vision method for detecting and segmenting objects in three-dimensional imagery (StartDist-3D) is applied to X-ray micro-computed tomography scans of oilseed rape (*Brassica napus*) mature pods.

**Results:**

With a relatively minimal training effort, this fine-tuned StarDist-3D model accurately detected (Validation F1-score = 96.3%,Testing F1-score = 99.3%) and predicted the shape (mean matched score = 90%) of seeds.

**Discussion:**

This method then allowed rapid extraction of data on the number, size, shape, seed spacing and seed location in specific valves that can be integrated into models of plant development or crop yield. Additionally, the fine-tuned StarDist-3D provides an efficient way to create a dataset of segmented images of individual seeds that could be used to further explore the factors affecting seed development, abortion and maturation synchrony within the pod. There is also potential for the fine-tuned Stardist-3D method to be applied to imagery of seeds from other plant species, as well as imagery of similarly shaped plant structures such as beans or wheat grains, provided the structures targeted for detection and segmentation can be described as star-convex polygons.

## 1 Introduction

The study of plant traits, such as plant architecture, growth, development, physiological or biochemical profiles is known as plant phenotyping. Identifying connections between plant genotype and phenotype is essential to advance our understanding of underlying developmental mechanisms in plant biology. With the recent rapid progression in functional genomics due to advances in high throughput sequencing, quantitative analyses of plant traits are of increasing relevance as it could allow for the links between genotype and phenotype to be explored in greater depth. For example, studies such as Genome-Wide Association Studies (GWAS) involve testing genetic variants across genotypes of a population to identify genotype-phenotype associations and provide essential information for plant breeding (Brachi et al., 2011; Alseekh et al., 2021). By obtaining insights on how genetics and environmental pressures lead to different phenotypic response in plants, more suitable and sustainable crops can be selected for growth in specific environments, as by identifying the genetic basis of phenotypic variation a better understanding of the factors driving plant adaptation and stress tolerance could be achieved.

The need to better characterize plant developmental growth stages and monitor traits that affect yield has led to an increased demand for and collection of high-throughput, high resolution plant image datasets (Costa et al., 2019). Analysis of such image datasets could allow for a more detailed understanding of dynamic developmental changes and for phenotypic traits to be measured in a non-destructive manner in comparison to current commonly used manual phenotyping methods. However, manual analysis of these image datasets can also be time-consuming, inconsistent, and requires expert observers. Therefore, developing reliable and efficient methods for automated extraction of phenotype data from plant images is crucial.

Image analysis pipelines for easy phenotyping have recently become more widely available such as those for measuring leaf area, leaf growth and root traits (Bours et al., 2012; Easlon & Bloom, 2014; Seethepalli et al., 2021). Although image acquisition is relatively straightforward, image analysis is plagued by a number of bottlenecks. In most cases, image thresholding and data extraction is still laborious and requires manual input. There also is a lack of consistency with regards to image acquisition between different days or between laboratories, which hampers the reliable extraction of phenotypic traits. Moreover, the automated images acquired in the agricultural sciences are driven by specific biological hypotheses, and the downstream pipelines typically are purpose-built and not compatible to other research areas, and often not free or easy to use.

In recent years, the study of plant organs and tissue development has been focused on the use of confocal microscopy, where 2D and 3D information is obtained by optical sectioning and the use of fluorescent markers. However, this is limited by the thickness of the sample being studied and the availability of suitable markers. A very valuable non-invasive and cost-effective 3D imaging technique for detecting and quantifying internal structures in a non-destructive manner without the necessity of using stains is X-ray micro-computed tomography (µCT), which is based on differential X-ray attenuation by biological materials. µCT scanners were developed mainly for medical purposes, and are not widely used in plant sciences (Dhondt et al., 2010; Pajor et al., 2013; Piovesan et al., 2021). To date, there has been limited application of this method to visualize above-ground plant structures because of the low attenuation density that these tissues present, resulting in images with low contrast (Pajor et al., 2013). However, recent improvements in scan resolution, quality and scan speed of current state-of-the-art µCT scanners present an opportunity to analyse these above-ground plant structures without the necessity of fixing or staining them. The µCT scanner has recently been used for the analysis of different plant tissues and organs, such as seeds, fruits, rice and wheat spikes flowers and leaves traits (Rousseau et al., 2015; Hughes et al., 2017; Tracy et al., 2017; Mathers et al., 2018; Schneider et al., 2018; Xiong et al., 2019; Gargiulo et al., 2020; Hu et al., 2020; Kunishima et al., 2020; Liu et al., 2020; Narisetti et al., 2020). These advances make this a promising technique to study complex plant traits, such as the internal structure of opaque mature pods without requiring destructive dissection methods. The resulting images have a higher resolution than those generated using other techniques such as light boxes or a light sheet confocal.

Although µCT scanning is a valuable tool for obtaining high-resolution images, advanced computational skills are required to develop automated data extraction pipelines from these images. This study aims to improve our understanding of seed biology and its related traits in the *Brassica napus* crop. For this purpose, images of mature seed pods were acquired, and data relating to the seed number per pod (SNPP) and seed area, as well as pod length were semi-automatically extracted. Although counting SNPP manually is quite easy, it is time consuming, and further data must be manually processed. When obtaining these data for GWAS studies with 100 individuals in a population, several hundreds of images need to be processed, resulting in an arduous and non-straightforward task. Moreover, more specific and biologically important information such as the position of the seeds in different pod valves and their relative spacing, is difficult to obtain. Therefore, we are interested in applying machine learning methods to assess whether this would allow us to generate a straightforward automated pipeline with minimal pre-processing for data extraction of phenotypic measurements from 3D µCT pod image data, including the number, size and shape of seeds, as well as their spatial arrangement relative to each other and to other pod structures.

In order to automatically extract valuable phenotypic measurements from 3D µCT pod image data, the first step required is to locate all individual seeds within a 3D volume. In machine learning, this is referred to as an object detection task, and can typically be achieved using models trained to recognize the target object (in this case *B. napus* seeds) and output the centre-point and/or a bounding box for each detection (Weigert et al., 2020). To extract data on seed size and shape, each pixel in the 3D volume needs to be labelled as either seed or background. Machine learning models designed to perform semantic segmentation have been shown to be able to achieve this for 3D volume data with high accuracy, but do not discern between individual objects meaning they cannot provide information on seed number and location (Alalwan et al., 2021; Kar et al., 2021; Sodjinou et al., 2022). Therefore, an instance segmentation method that allows for both detection of multiple distinct objects, and that outputs both the number and location of seeds as well as a separate labelled mask capturing the shape and size of each seed is needed to achieve the goal of automatically extracting phenotype data from 3D µCT pod images (Lin et al., 2021; Wang et al., 2022).

Many automated instance segmentation methods have been proposed to process the increasingly large 3D volume datasets modern imaging instruments such as µCT scanners and microscopes are capable of producing (Meijering, 2012). These include non-machine learning approaches such as methods watershed transform-based morphological methods (Beucher & Meyer, 1993; Lotufo et al., 2002; Cheng & Rajapakse, 2009), graph-cut based optimization (Boykov & Funka-Lea, 2006), and thresholding or pixel-grouping using connected component analysis (Majanga & Viriri, 2021), as well as recent methods based on deep learning have been demonstrated to significantly improve the accuracy of instance segmentation predictions for images of biological specimens (Van Valen et al., 2016; He et al., 2017; Xie et al., 2018). These methods can be broadly sorted into two categories; methods in which semantic segmentation is performed first and pixels are then grouped into distinct objects (Çicek et al., 2016; Caicedo et al., 2019), and methods in which bounding boxes for individual objects are first predicted and then semantic segmentation is performed for each detected object (He et al., 2017; Xu et al., 2018; Zhao et al., 2018). However, despite the increased performance demonstrated by these deep learning methods in comparison to thresholding, watershed, and graph-cut optimization methods, they often still produce inaccurate results when used to predict the location and segment the individual shape of densely-packed objects, similar to the close positioning of seeds within the *B. napus* pod µCT image dataset (Schmidt et al., 2018).

StarDist-3D is an automated object detection and segmentation approach that was recently used to identify and examine the size, shape and spatial arrangement of individual cell nuclei in volumetric (3D) fluorescence microscopy images. It exhibits a high degree of accuracy in terms of both the predicted counts and shape of cell nuclei compared to other contemporary approaches such as U-Net and IFT-Watershed (Lotufo et al., 2002; Çicek et al., 2016; Schmidt et al., 2018; Weigert et al., 2020). The method uses a neural network to predict whether each pixel in a 3D volume is part of an individual target object, and to predict the distance to the object boundary using along several radial directions, defined based on spherical Fibonnaci lattice (Weigert et al., 2020). Thus, the shape of detected objects is predicted as a star-convex polygon, with increasing detail in the fluctuations along the surface of the object rendered when a higher number of Fibonnaci rays is used, allowing the anisotropy, or variation in shape of the target objects along different axial direction, of predicted objects to be more accurately reconstructed (Weigert et al., 2020).

It was determined that it would be potentially advantageous to apply the StarDist-3D approach to 3D µCT images of *B. napus* pods as the method was demonstrated to yield high accuracy in terms of both detection and segmentation for objects in close proximity to each other, as *B. napus* seeds are often observed to be tightly packed within pods (Weigert et al., 2020). Another potential advantage of the method was that it incorporates a computationally efficient non-maximum suppression (*nms*) process that reduces the likelihood of detecting the same object multiple times by suppressing detections with low confidence where the boundaries of detections with high confidence overlap (Schmidt et al., 2018; Weigert et al., 2020). Additionally, the StarDist-3D approach requires a relatively small amount of training data as it has been pre-trained to detect and segment a generalized variety of star-convex polygonal shapes. It is capable of quickly processing typically large 3D images, and the model outputs can easily be passed to state-of-the-art open-source toolkits for image analysis to extract specific data on the location, spatial arrangement, and 3D shape of predicted seeds (van der Walt et al., 2014; Gostick et al., 2019). Although the StarDist-3D has previously only been applied to cell microscopy images, it was posited that the method could be applied to any 3D volumetric image dataset regardless of sensor type or scale provided the target objects could be described as star-convex polyhedral. As *B. napus* seeds tend to be rounded or oval in shape, with slight variations or asymmetry along different axial directions, it was likely that the seeds could be appropriately described as star-convex polyhedra. Therefore, in this manuscript we explore the accuracy of detection and segmentation of a StarDist-3D model fine-tuned on 3D µCT images of *B. napus* pods, along with investigation of extraction of data on seed size, shape and spatial arrangement from the model outputs which could provide important biological information to improve models pertaining to plant development and crop yield.

## 2 Materials and methods

### 2.1 Plant growth conditions and data collection

A *B. napus* diversity set population with ninety-six genotypes was grown as in Siles et al. (2021). The seeds were germinated in P24 trays with John Innes Cereal Mix and once they presented four true leaves, they were transferred to a vernalization room with an 8 h photoperiod at 4°C day/night for 8 weeks. Each plant was re-potted in a 2 L pot in John Innes Cereal Mix. Each genotype had five biological replicates and once out of vernalization, all plants were grown in two glasshouse compartments in long-day conditions (16 h photoperiod) at 18°C day/15° night (600w SON-T, high pressure sodium lighting) at a density of 12 pots per m^2^. Once the plants were fully dry and mature, the first five dry pods on the main inflorescence were ignored, and the next three developed pods were collected for scanning. To avoid pod shattering the pods were sprayed with Prism Clear Glaze (Loxley Arts, Sheffield, UK).

For each genotype, three fully dried pods were placed in plastic holders (34mm x 110mm) and packing peanuts were used to keep the samples in place while scanning. The pedicel was cut with a scalpel before placing the pods into the plastic holders. If the pods were too tall to fit in the holders, they were cut into two pieces and were separately scanned. Twelve holders were loaded into the sample changing carousel of a μCT100 scanner (Scanco Medical, Switzerland). This scanner has a cone beam X-ray source with power ranging from 20 to 100 kVp (pre-set and calibrated for 45, 55, 70, 90 kVp) and a detector consisting of 3072 × 400 elements (48 µm pitch) and a maximum resolution of 1.25 µm. Pods were scanned with the X-ray power set at 45 kVp, 200 µA, 9W, with an integration time of 200 ms.

### 2.2 Image dataset description

Images were retrieved from the proprietary Scanco microCT file type format (.ISQ), which contained single-pixel width two-dimensional (2D) trans-axial projections, or ‘slices’, that together formed stacks depicting an entire pod as three-dimensional (3D) volumes. Thirty-two distinct 3D volumes were included in the experiment dataset, each containing a single entire *B. napus* pod. All 2D trans-axial (XY) slices were 512 × 512 pixels, therefore the height and width of all 3D volumes was also 512 pixels. Individual 3D volumes varied in length from 505 to 1397 slices, with a total of 29,871 slices in the experimental dataset. The total dataset contained 471 seeds.

The total dataset was split into a model training and validation dataset comprised of 13 3D volumes, 12,475 2D slices and 262 seeds and a model testing dataset containing comprised of 19 3D volumes, 17,396 2D slices and 209 seeds. This split was decided upon due to the uneven number of seeds in each seed pod, with the training and validation dataset containing 262(56%) of seeds and the testing dataset containing 209(44%) of seed. Another factor impacting the split of data was that intact 3D volumes of entire seedpods needed to be used for testing, to demonstrate that reliable seed detection and segmentation could be achieved on the original imagery without any pre-processing. Conversely, Weigert et al. (2020) demonstrated that more accurate results could be obtained in a computationally efficient manner by training a StarDist-3D on smaller sub-volumes of the original 3D volume data containing objects of interest, in this case sub-volumes containing at least one entire seed. The 3D volumes in the model training and validation dataset were therefore comprised of 138 small sub-volumes of stacked 2D slices containing a single seed, or multiple seeds in instances where seeds occupied some of the same 2D slices. These sub-volumes ranged in size between 24 to 84 2D slices depending on the size of the single seed or multiple overlapping seeds contained within. This sub-division was carried out in order to ensure a mixture of seeds from different seed pods could be used for model training and validation. 117 sub-volumes containing 220 seeds were randomly sorted into the final ‘training’ dataset, and 21 volumes containing 42 seeds were sorted into the final ‘validation’ dataset.

### 2.3 Image pre-processing and annotation

All 3D volumes contained in the experimental dataset were batch converted from their original.ISQ format into.TIF stacks using BoneJ plugin (Domander et al., 2021) for Fiji ImageJ software version 2.9.0 (Schindelin et al., 2012). All 262 seeds contained within the 138 3D sub-volumes comprising the ‘training’ and ‘validation’ datasets were then manually annotated using Fiji and the Labkit plugin (Schindelin et al., 2012; Arzt et al., 2022). Sub-volumes were converted from XYZ format to XYT timeseries using the ‘re-order hyperstack’ function provided by Fiji. Labelled masks the entire area covered by the seed in each 2D slice were then created using Labkit, with the same label applied to all pixels contained within a single seed as it appeared across multiple slices. 3D masks of the entire shape (interior and exterior) of each seed were then created by stacking the slices with 2D label masks. During this annotation process the true number of seeds within each seed pod was recorded by manually counting the seeds within each 3D volume.

### 2.4 Seeds as star-convex polygons

To determine whether the shape of *B. napus* seeds could be appropriately described by star-convex polygons, the accuracy of reconstruction of ground truth labels for a small subset of 10 3D sub-volumes from the ‘training’ dataset was explored. Accuracy of reconstructed seeds was assessed based on the mean intersection-over-union (IoU) of ground-truth seed labels compared to 3D star-convex polyhedra shape representations of the seed, predicted using the method described by Weigert et al. (2020) in which for each pixel inside a seed the distance to the object boundary is calculated along a fixed set of rays that are approximately evenly distributed on an ellipsoid representative of the seeds within the dataset (see Weigert et al., 2020 eq. 1). The sets of rays used in seed reconstruction were calculated as Fibonacci rays, defined using the method described by Weigert et al. (2020), which were shown to be more accurate for reconstruction of 3D star-convex polyhedra than equidistant distributed rays and allowed for the potential anisotropy of seed to be taken into account. Reconstruction accuracy was investigated using a varying number of Fibonacci rays (8, 16, 32, 64, 96, and 128), as although Weigert et al. (2020) found a set of at least 64 rays was necessary to achieve accurate reconstruction of shape for cell nuclei, they suggested accurate reconstruction of less anisotropic or densely-packed objects may be possible with a smaller set of rays which would allow for less computational resources to be used in shape prediction.

### 2.5 Model training and validation

A StarDist-3D model with a U-Net backbone (Çicek et al., 2016) was trained to detect and segment individual *B. napus* seeds in 3D µCT sub-volumes from the labelled ‘training’ dataset using the pipeline described by Weigert et al. (2020). Model training was performed using a Google Colab runtime with 25.46 GB and a single GPU (Bisong, 2019). The StarDist-3D model was configured to use 96 Fibonacci rays in shape reconstruction, and to take into account the mean empirical anisotropy, of all labelled seeds in the dataset along each axis as calculated using the method described by Weigert et al., 2020 (X-axis = 1.103448275862069, Y-axis anisotropy = 1.032258064516129, Z-axis anisotropy = 1.0). The training patch size, referring to the size of the tiled portion of the 3D sub-volumes in the ‘training’ within view of the neural network at any one time, was set to Z = 24, X= 96, and Y = 96 and training batch size set to 2. Training ran for 400 epochs with 100 steps per epoch and took 1.36 hours to complete (123ms/step).

Model validation was then performed by using the fine-tuned StarDist-3D algorithm to predict seed labels for all 3D µCT sub-volumes from the ‘validation’ dataset, which were then compared to the number and shape of seeds manually counted and labelled during annotation. Accuracy of seed detection and segmentation was then quantified for various levels of threshold τ, defined as the IoU between the predicted label and ground-truth label for each seed. The value of τ ranged between 0, where even a very slight overlap between predicted seeds and actual seeds counted as correctly predicted, and 1, where only predicted seed labels with pixel-perfect overlap with ground-truth labels counted as correctly predicted (Weigert et al., 2020).

Object detection accuracy was measured using the number of true positive results (TP), or number manually counted and labelled seeds that were correctly detected seeds, the number of false negative results (FN), or the number of manually counted and labelled seeds that were missed, the number of false positive results (FP), or number of objects other than seeds than were detected, recall, precision and F1-score. Recall related to the fraction of relevant objects that were successfully detected and was defined as:


$$Recall=\frac{TP}{TP+FN}$$

Precision related to the fraction of all detected objects that were relevant and was defined as:


$$Precision=\frac{TP}{TP+FP}$$

F1-score related to the harmonic mean of precision and recall, with the impact of precision and recall being given equal weight. F1-score was defined as:


$$F=2\times \frac{Precision\;\times Recall}{Precision+Recall}$$

The accuracy of seed segmentation, or the accuracy of seed size and shape prediction, for the validation dataset was determined based on the mean matched score, defined as the mean IoU between the predicted and actual shape of true positive results, the mean true score, defined as the mean IoU between the predicted and actual shape of true positive results normalised by the total number of ground-truth labelled seeds, and panoptic quality, as defined in Eq.1 of Kirillov et al., 2019.

StarDist-3D models allow for specification of two values, the τ-threshold and the *nms*-threshold to optimize model output (Schmidt et al., 2018; Weigert et al., 2020). The τ-threshold refers to the minimum intersection-over-union between pairs of predicted and ground-truthed seeds required for detections to be classified as true positives, and can be set at 0.1 interval levels between 0.1 and 1 with 0.1 indicating a 10% overlap in the pixels within the predicted shape of a seed and the ground-truthed label and 1 respreseting a 100% overlap (Schmidt et al., 2018; Weigert et al., 2020). The *nms*-threshold, refers to the level of non-maximum suppression applied to the results of object detection and instance segmentation to prune the number of predicted star-convex polyhedra in ideally retain a single predicted shape for each true object, in this case each seed, within an image. The *nms*-threshold can be set at 0.1 interval levels between 0 and 1 with higher levels indicating more aggressive pruning of predicted shapes which therefore leads to fewer detections in the final model output. Therefore a higher *nms*-threshold is valuable in cases where the number of false positives expected in unfiltered model predictions is high. Both the τ-threshold and the *nms*-threshold for the fine-tuned StarDist-3D algorithm were set to optimal values based on the ‘validation’ dataset using the ‘optimize_thresholds’ function of StarDist (Schmidt et al., 2018).

### 2.6 Model testing and outputs

Testing of the fine-tuned StarDist-3D algorithm was carried out using the ‘test’ dataset, which was kept separate from model training and validation. Model testing was also carried out using the same Google Colab instance as model training and validation. Prediction, including both detection and segmentation of seeds took on average 1 minute 24 seconds to complete for a single complete 3D µCT volume containing a whole *B. napus* pod. Accuracy of seed detection was quantified using the same metrics as model validation, with the predicted number and location of seeds compared to the true number of locations of seeds in each image.

The output of prediction for the fine-tuned StarDist-3D algorithm were 3D numpy array volume containing labels depicting the predicted shape of seeds for each 3D µCT volume of a whole *B. napus* pod. The number and location of seeds including both bounding box and centroid coordinates on the Z, Y and X axis of the 3D volume could then be retrieved using the ‘regionprops’ and ‘regionprops_table’ functions of ‘scikit-image’ (van der Walt et al., 2014), an open-source python image analysis package (**Table 1**). Measurements of the 3D shape of seeds could also be extracted from the fine-tuned StarDist-3D model predictions using the ‘regionprops_3D’ and ‘props_to_Dataframe’ functions of ‘porespy’ (Gostick et al., 2019), an open-source python toolset for extracting data from 3D images of porous materials (**Table 2**). Seed size and shape metrics extracted using porespy functions included:

- Volume – the predicted volume of a detected seed in number of voxels
- Bounding box volume – the volume of the rectangular 3D bounding box containing a detected seed in number of voxels
- Sphericity – the ratio of the area of a sphere with the same volume as a detected seed to the predicted surface area of the same detected seed
- Surface area – the predicted surface area of a detected seed calculated using a reconstructed mesh of the surface contour of the seed
- Convex volume – number of pixels in the predicted convex hull image of a detected seed
- Equivalent diameter – the diameter of a circle with the same area as a detected seed
- Extent – ratio of pixels within the predicted shape of a detected seed to the total pixels within the 3D rectangular bounding box containing the seed
- Major axis length - the width of the thickest part of the seed, measured as a straight line through an ellipse that has the same normalized second central moments as the detected seed
- Minor axis length - the width of the thinnest part of the seed, measured as a straight line through an ellipse that has the same normalized second central moments as the detected seed
- Solidity – ratio of number of pixels within the predicted shape of a detected seed to number of pixels within the convex hull images of the same detected seed

**Table 1:** Example of data extracted on *Brassica napus* seed number per pod and seed location derived from fine-tuned StarDist-3D algorithm predictions.

Seed ID Number	Z-axis minimum	Y-axis minimum	X-axis minimum	Z-axis maximum	Y-axis maximum	X-axis maximum	Z-axis centroid	Y-axis centroid	X-axis centroid
***1***	354	258	221	387	293	257	370.3969	274.8411	238.3993
***2***	248	282	213	281	318	248	264.5435	299.7615	229.9509
***3***	504	264	214	538	299	250	520.8316	280.5683	231.2285
***4***	293	258	219	326	293	255	308.6688	274.7142	236.1587
***5***	185	271	206	215	304	239	199.2717	286.0577	221.4716

**Table 2:** Example of data extracted on *Brassica napus* seed size and shape derived from fine-tuned StarDist-3D algorithm predictions.

Seed ID Number	Volume	Bounding box volume	Sphericity	Surface area	Convex volume	Equivalent diameter	Extent	Major axis length	Minor axis length	Solidity
*1*	23237	41580	0.886865	4440.313	24538	35.40466	0.55885	45.38723	44.23421	0.94698
*2*	23379	41580	0.89321	4426.713	24627	35.47664	0.562266	45.43497	44.46307	0.949324
*3*	23344	42840	0.870115	4539.668	24680	35.45892	0.544911	45.77541	44.01394	0.945867
*4*	21921	41580	0.82114	4612.895	23238	34.72327	0.527201	44.78899	43.01572	0.943326
*5*	19451	32670	0.95953	3645.191	20476	33.36679	0.595378	43.07271	41.68581	0.949941

**Figure 1:**
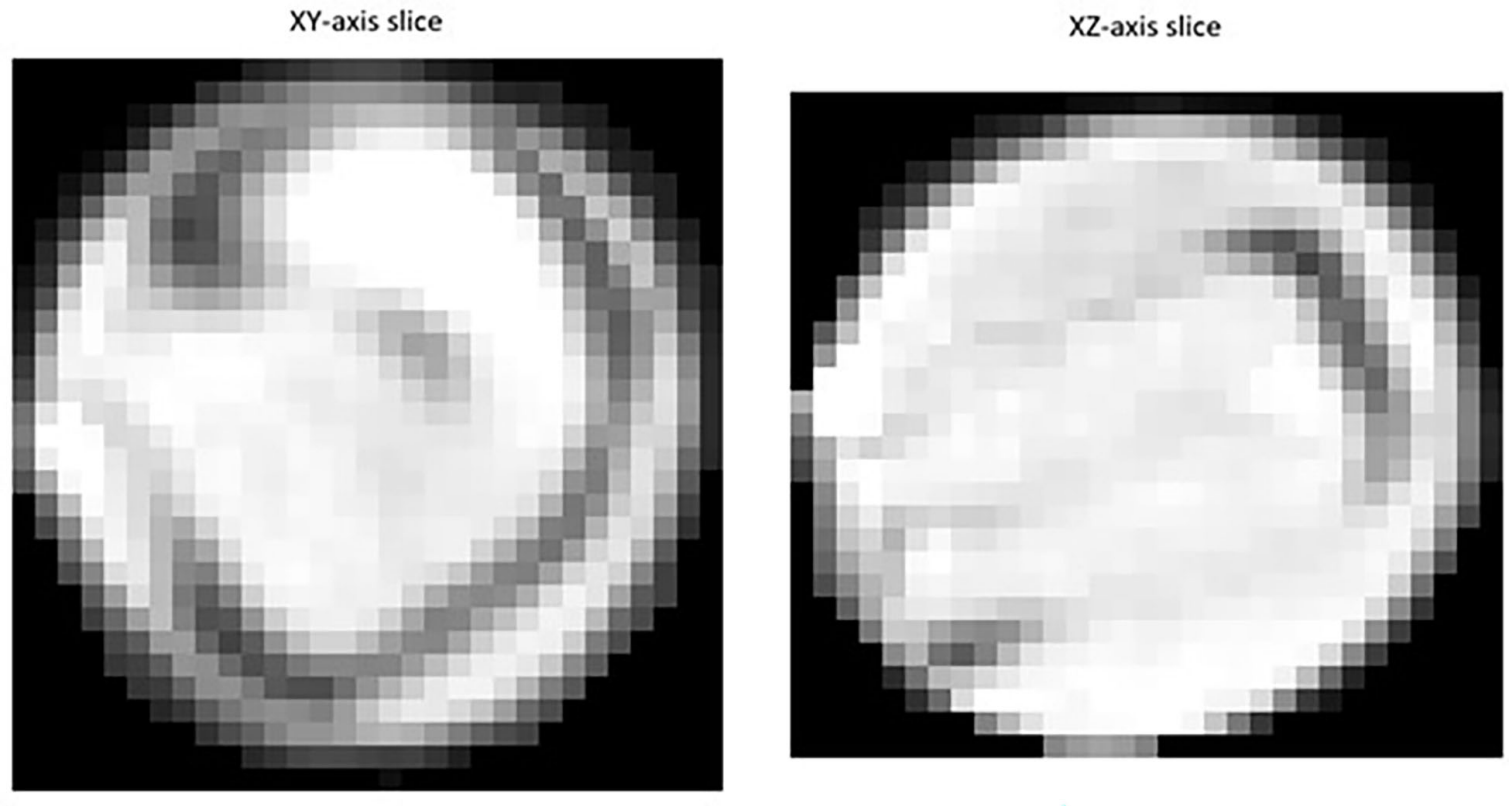
Example of a 2D slice images extracted for individual *Brassica napus* seeds detected and segmented with a fine-tuned StarDist-3D algorithm from 3D micro-computed tomography.

Segmented images of individual detected seeds can also be exported. 3D volume images of individual seeds can be converted into numpy arrays and saved for further investigation using the open-source ‘numpy’ python package (Harris et al., 2020). As shown in **Figure 1**, individual 2D trans-axial slices showing a cross-section of detected seeds on both the XY and XZ axis can also be viewed and exported using the ‘intensity_image’ function of scikit-image (van der Walt et al., 2014).

### 2.7 Automated seed sorting by pod valve

**Figure 2:**
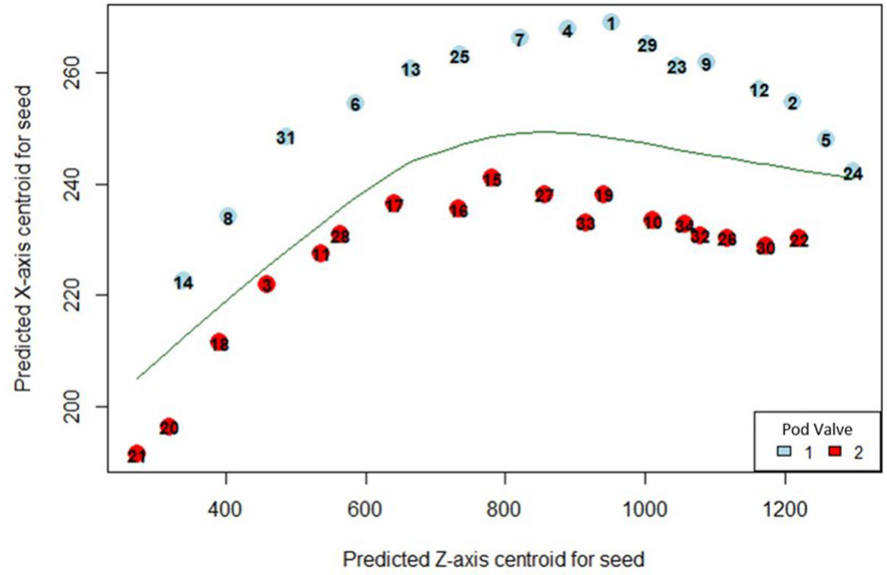
Predicted position of automatically detected *Brassica napus* seeds in pod valves using automated lowess regression. Points on the graph indicate the XZ centroid of detected seeds with a unique seed identification number and are coloured based on whether they were predicted to be positioned in valve 1 (blue) or valve 2 (red).

**Figure 3:**
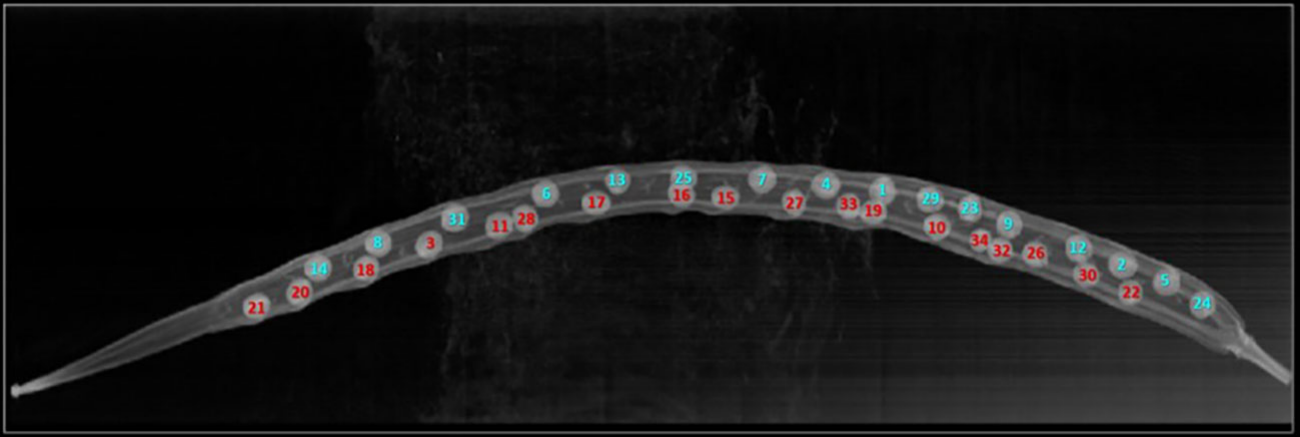
Original XZ slice image of a *Brassica napus* pod (same pod for which valve predicted was performed as shown in **Figure 4**). Seeds are marked with a unique identification number matching **Figure 4** and are coloured by whether they were confirmed to be positioned in valve 1 (blue) or valve 2 (red) through manual analysis.

In order to predict the valve in which the detected seeds were situated within the pod, coordinates of seeds detected with the fine-tuned StarDist-3D algorithm were converted to.csv format using the ‘pandas’ python package (McKinney, 2010) in order to allow loading into RStudio (RStudio Team, 2020). A locally weighted scatterplot smoothing (lowess) regression line was then fit to the XZ axis centroids of detected seeds using the ‘lowess’ function of the ‘gplots’ package in R (Cleveland 1979; Cleveland 1981; Warnes et al., 2005; R Core Team, 2018). The lowess regression line was then used to predict the division between the two valves of the seed pod, serving as a simplified reconstruction of the pod pseudoseptum, which is the membrane that separates both valves (**Figure 2**). The XZ centroid was used as all pods were arranged the same way during image collection so that the XZ plane displayed a cross-section of the pod with seeds sitting in one of two valves separated by the pod pseudoseptum, with the pod beak on the left and the pod pedicel on the right (**Figure 3**). In cases where pods contained less than or equal to 5 seeds, the smoother span (*f*), or proportion of points influencing the smooth at each value for the lowess regression line was set to *f* = 1. For seed pods containing greater than 5 seeds the default value of *f* provide by the ‘lowess’ function was used. The vertical distance between the XZ centroid of detected seeds and lowess regression line was then calculated and seeds found to be above the lowess regression line were determined to belong to ‘valve 1’ while seeds below the line were determined to belong to ‘valve 2’. The sequence number for detected seeds in each valve from pod beak to pedicel, and the distance between sequential seeds in each valve could then be calculated and added to the.csv data of seed coordinates for each seed pod. The base R function ‘for’ was used to create a looping script to automate the prediction of valve and calculation of valve related metrics for all seeds in all seedpods and on average it took 70 milliseconds to complete valve prediction and valve-related metric extraction for an entire pod using a single CPU.

**Figure 4:**
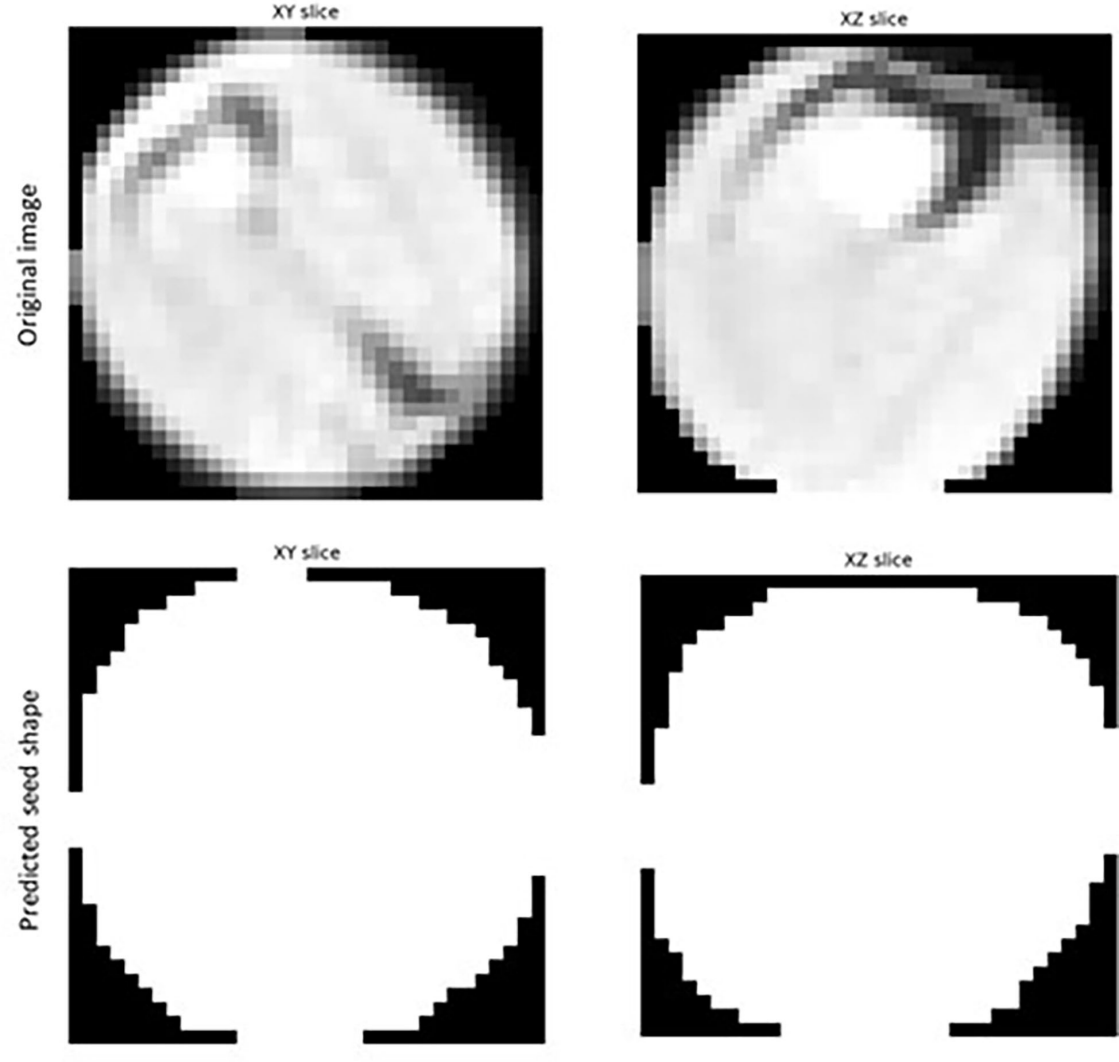
Example of segmentation results for individual *Brassica napus* seeds detected in 3D micro-computed tomography scans of seed pods from the ‘test’ dataset.

## 3 Results

### 3.1 Accuracy of reconstruction of seed labels as star-convex polygons

**Figure 5:**
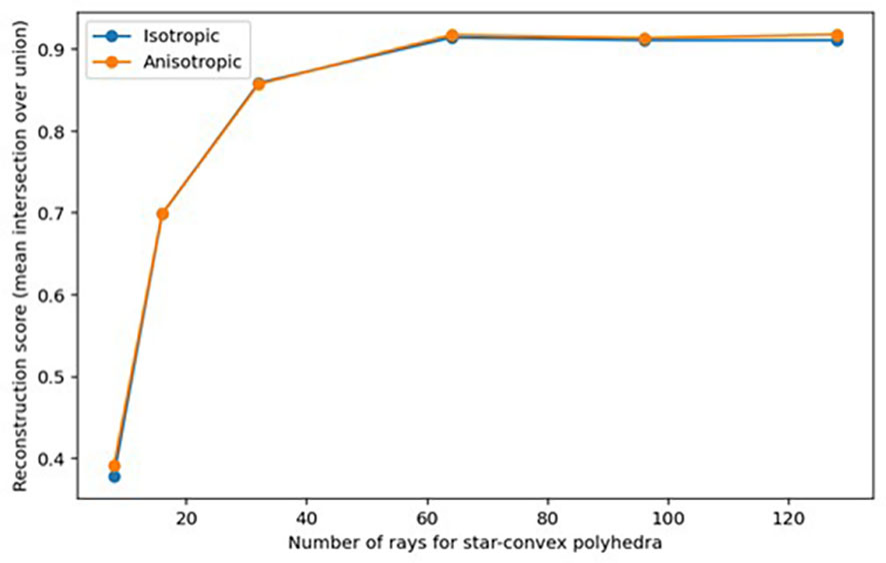
Reconstruction accuracy (mean IoU) of ground-truth labelled *Brassica napus* seeds when using different unit Fibonacci rays.

**Figure 6:**
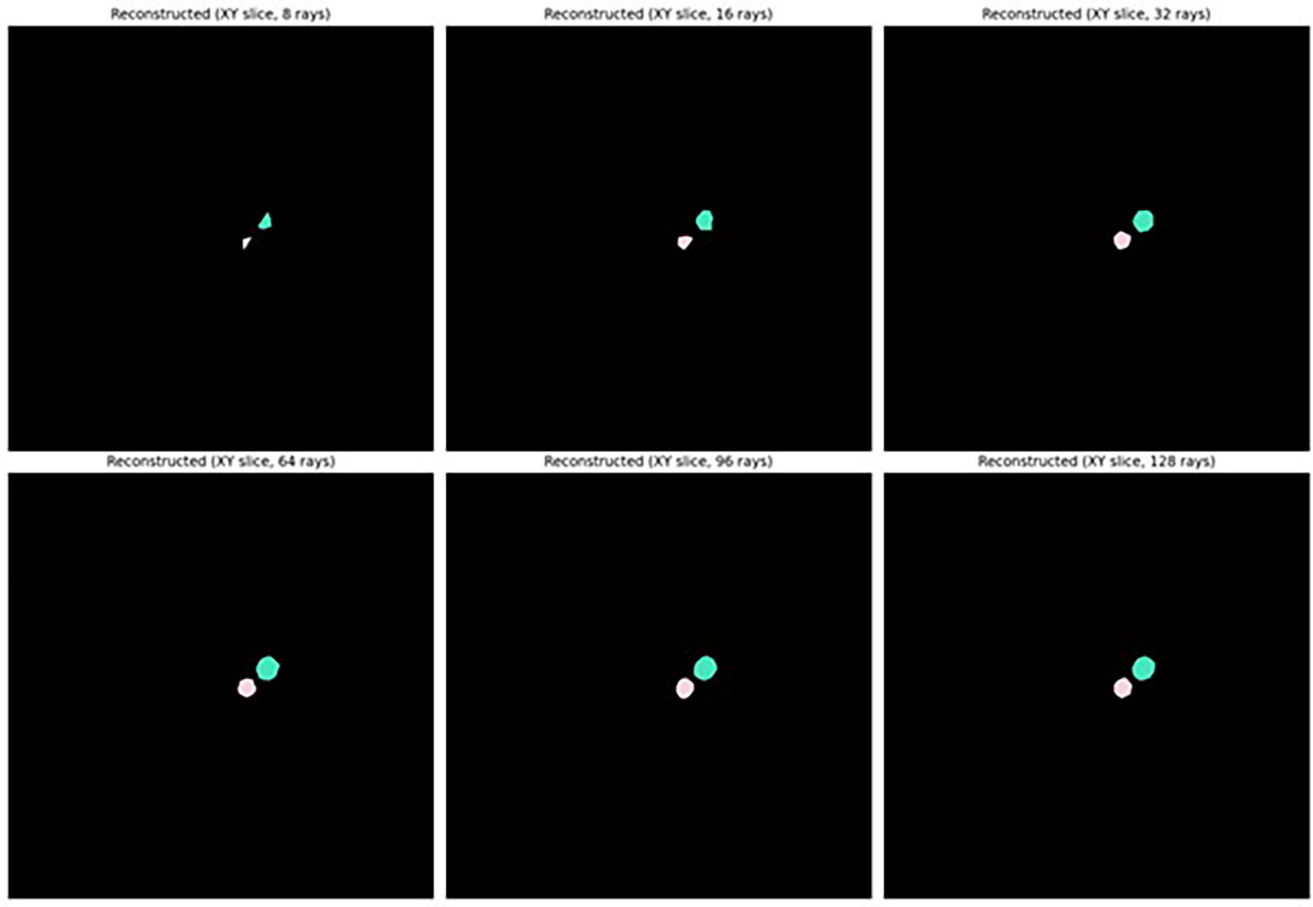
Reconstructed shape of *Brassica napus* seeds using different numbers of Fibonacci rays with anisotropy of seeds taken into account.

As shown in **Figure 5**, sufficiently accurate reconstruction (greater than 0.8 mean IoU) of labelled seeds was achieved with as few as 32 rays with or without taking anisotropy into account, and the highest reconstruction accuracy (greater than 0.9 mean IoU) was achieved when 64 rays or more were used. It was therefore determined that it was appropriate to describe the shape of seeds as star-convex polyhedral and to proceed with training a StarDist-3D for detection and segmentation of seeds. It was also decided that reconstruction with anisotropy should be used that would more easily allow application of the workflow described in this paper to images of seeds or other star convex plant structures that may be more irregular in shape. Example reconstruction of seed shape with anisotropy taken into account is demonstrated in **Figure 6**. See **Supplementary Figure 1** for seed reconstruction without anisotropy.

### 3.2 Seed detection and segmentation model validation

The trained StarDist-3D model was tested with different certainty thresholds (τ) to predict the number and shape of seeds within a pod. For τ = 0.1 to 0.8, 39 of the 42 seeds contained within the validation data were detected using the fine-tuned StarDist-3D algorithm and there were no false positive results (**Table 3**). Therefore, the recall rate (the actual number of seeds in the image that were successfully detected) was 92.9%, the precision rate (the number of detected objects in the image that were seeds) was 100%, and F1-score was 96.3% for the validation dataset across this range of τ (**Table 3**).

**Table 3:** Accuracy metrics for automated detection and segmentation of *Brassica napus* seeds in 3D micro-computed tomography scans from the ‘validation’ dataset across several intersection-over-union thresholds τ.

Threshold τ	0.1	0.2	0.3	0.4	0.5	0.6	0.7	0.8	0.9
Detection Metrics									
***True Number of Seeds***	*42*	*42*	*42*	*42*	*42*	*42*	*42*	*42*	*42*
***Number of True Positives***	*39*	*39*	*39*	*39*	*39*	*39*	*39*	*39*	*25*
***Number of False Negatives***	*3*	*3*	*3*	*3*	*3*	*3*	*3*	*3*	*17*
***Number of False Positives***	*0*	*0*	*0*	*0*	*0*	*0*	*0*	*0*	*14*
***Precision***	*1.000*	*1.000*	*1.000*	*1.000*	*1.000*	*1.000*	*1.000*	*1.000*	*0.641*
***Recall***	*0.929*	*0.929*	*0.929*	*0.929*	*0.929*	*0.929*	*0.929*	*0.929*	*0.595*
***F1-score***	*0.963*	*0.963*	*0.963*	*0.963*	*0.963*	*0.963*	*0.963*	*0.963*	*0.617*
Segmentation Metrics									
***Mean true score***	*0.836*	*0.836*	*0.836*	*0.836*	*0.836*	*0.836*	*0.836*	*0.836*	*0.543*
***Mean matched score***	*0.900*	*0.900*	*0.900*	*0.900*	*0.900*	*0.900*	*0.900*	*0.900*	*0.912*
***Panoptic quality***	*0.867*	*0.867*	*0.867*	*0.867*	*0.867*	*0.867*	*0.867*	*0.867*	*0.563*

**Figure 7:**
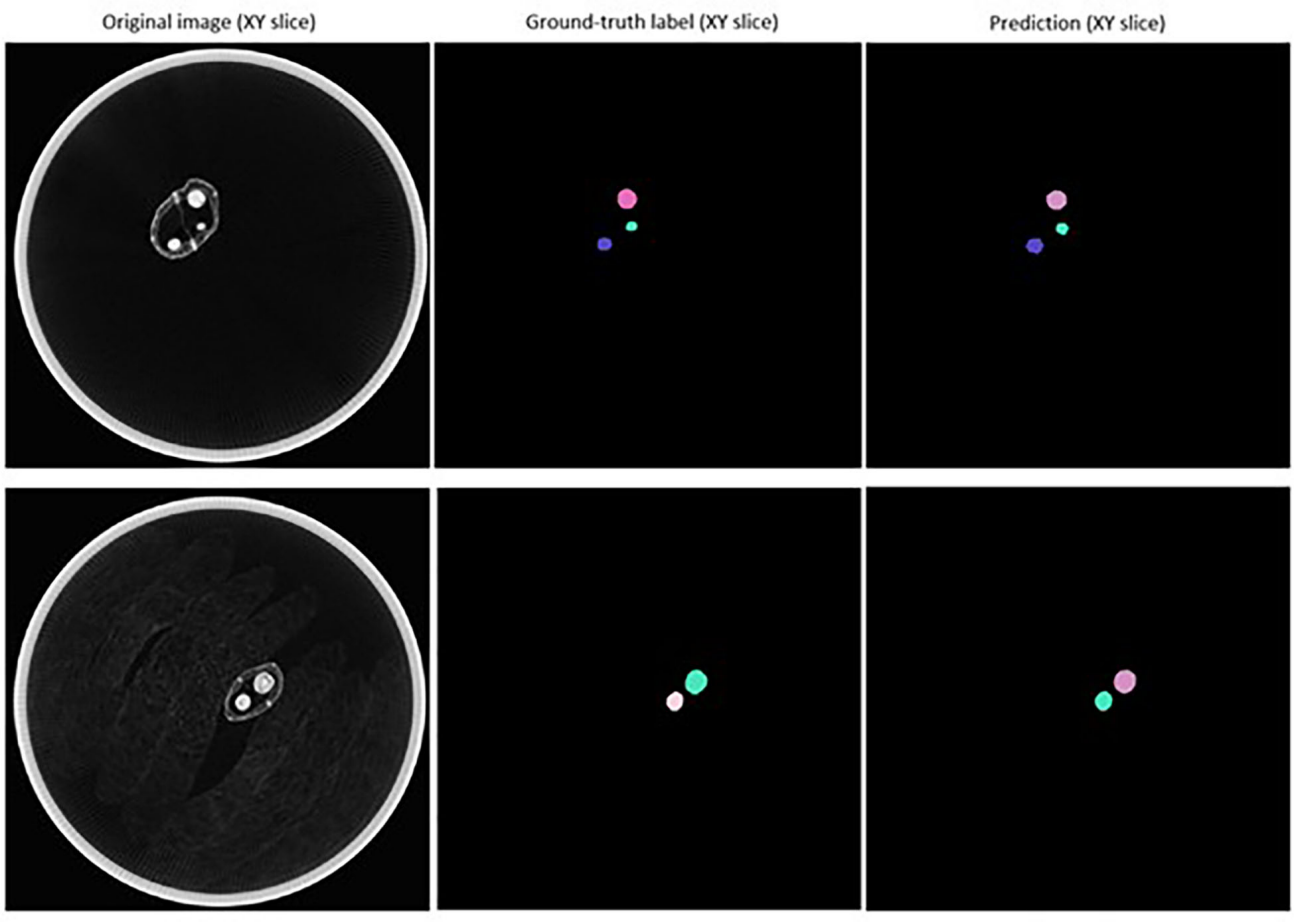
Example of segmentation results for *Brassica napus* seeds detected in 3D micro-computed tomography scans of seed pods from the ‘validation’ dataset (τ = 0.7).

The mean matched score when τ = 0.1 to 0.8 was 0.900, indicating a 90.0% overlap in pixels predicted to be a part of detected seeds with pixels known to be a part of ground-truth labelled seeds (**Table 3**). The mean true score (0.836) and panoptic quality (0.867) for this range of τ also suggested a high degree of overlap between the predicted and actual shape of detected seeds (**Table 3**). **Figure 7** displays example segmentation results for individual seeds detected with the fine-tuned StarDist-3D algorithm.

When τ was increased from 0.8 to 0.9 a slight increase in mean matched score from 0.900 to 0.912 occurred, but a large decrease in accuracy of both detection and segmentation as indicated by all other metrics was observed (**Table 3**). Setting thresholds of τ = 0.7 and *nms* = 0.4 resulted in the highest precision, recall and F1-score accuracy for seed detection and were therefore identified as optimal and incorporated into the fine-tuned StarDist-3D model used to perform prediction on novel pod data.

### 3.3 Seed detection and segmentation model testing

The true number of seeds contained within ‘test’ dataset was 209, while the total number of seeds predicted to be present within the ‘test’ images using the fine-tuned StarDist-3D algorithm was 208 (**Table 4**). One predicted seed was determined to be a pod pedicel incorrectly labelled as a seed and was recorded as a false positive result, while two seeds observed in the ‘test’ dataset were missed and recorded as false negative results. Therefore, the overall precision rate of the fine-tuned StarDist-3D algorithm when applied to the ‘test’ dataset was 99.52%, the overall recall rate was 99.04%, and the overall F-score was 99.28% (**Table 4**). Within individual pods the precision rate ranged from 95-100%, the recall rate ranged from 90.48-100%, and the F-score ranged from 95-100% (**Table 4**).

**Table 4:** Accuracy metrics for automated detection of *Brassica napus* seeds in 3D micro-computed tomography scans of pods from the ‘test’ dataset.

Seed Pod ID	True Number of Seeds	Predicted Number of Seeds	Number of True Positives	Number of False Positives	Number of False Negatives	Recall	Precision	F1-score
**C0007186**	5	5	5	0	0	100	100	100
**C0007197**	19	20	19	1	0	100	95	97.44
**C0007198**	4	4	4	0	0	100	100	100
**C0007205**	2	2	2	0	0	100	100	100
**C0007224**	34	34	34	0	0	100	100	100
**C0007226**	6	6	6	0	0	100	100	100
**C0007239**	8	8	8	0	0	100	100	100
**C0007256**	30	30	30	0	0	100	100	100
**C0007262**	2	2	2	0	0	100	100	100
**C0007269**	1	1	1	0	0	100	100	100
**C0007274**	34	34	34	0	0	100	100	100
**C0007299**	3	3	3	0	0	100	100	100
**C0007311**	15	15	15	0	0	100	100	100
**C0007420**	7	7	7	0	0	100	100	100
**C0007440**	21	19	19	0	2	90.48	100	95
**C0007456**	4	4	4	0	0	100	100	100
**C0007853**	6	6	6	0	0	100	100	100
**C0007864**	4	4	4	0	0	100	100	100
**C0007865**	4	4	4	0	0	100	100	100
**Total**	**209**	**208**	**207**	**1**	**2**	**99.04**	**99.52**	**99.28**

**Figure 8:**
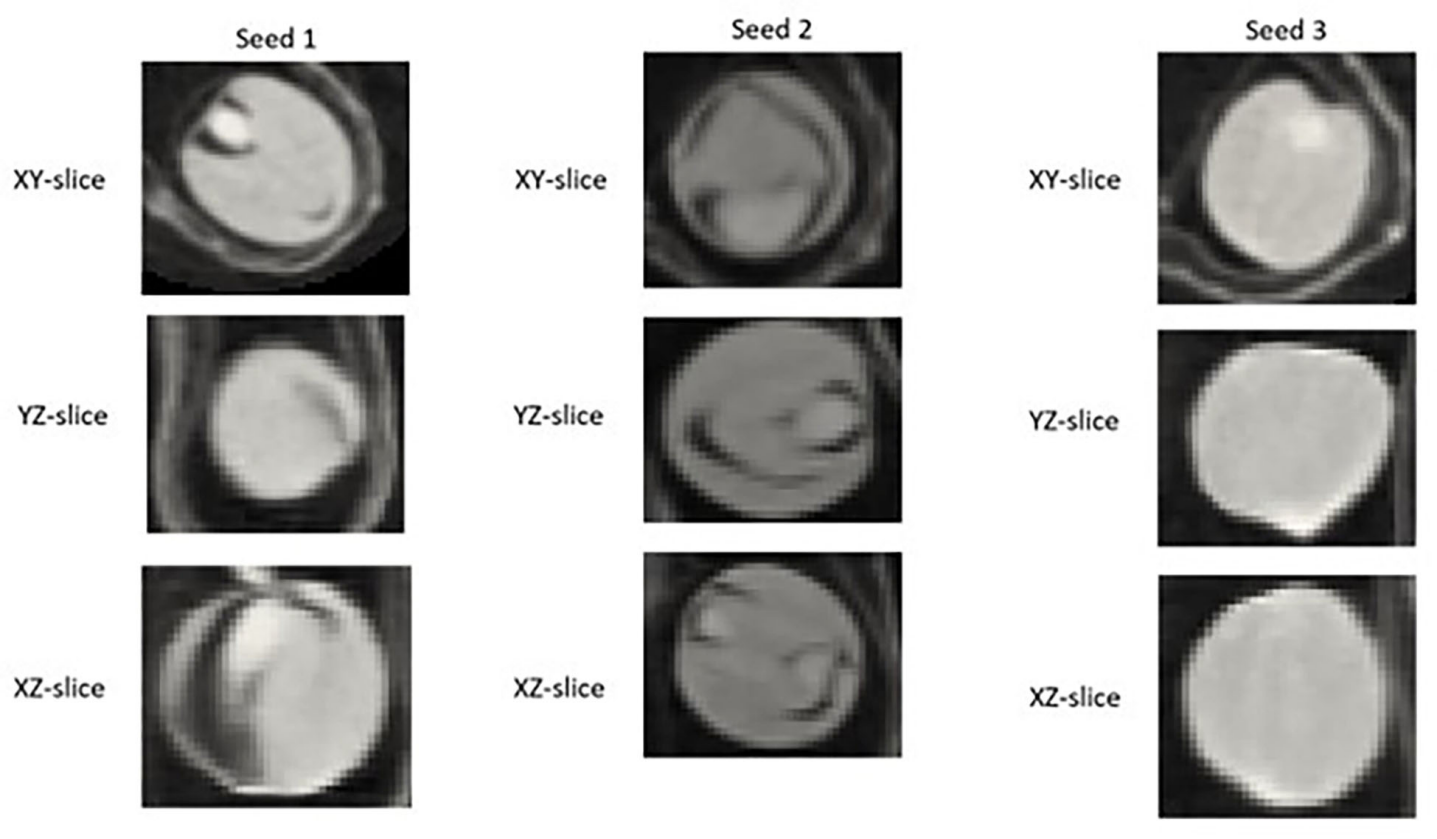
2D XY, YZ, and XZ slice images of individual *Brassica napus* seeds detected and segmented with a fine-tuned StarDist-3D algorithm from the same pod displaying variation in internal structure, particularly empty (black) space inside of seeds.

Example segmentation results for individual seeds detected in pod images from the ‘test’ dataset are displayed in **Figure 4**.** **A large degree of variance in the appearance of the interior of detected and segmented seeds, in particular the amount of empty space within the seed (depicted by pixels closer to black in colour) was observed, even within the same pod (**Figure 8**).

### 3.4 Automated seed sorting by valve

198 out of the 209 seeds contained in the ‘test’ dataset were sorted into the correct valve using the automated seed sorting method, resulting in an overall accuracy of 94.74% (**Table 5**). The percentage of correctly sorted seeds within a single pod ranged from 50-100%, with seed sorting accuracy below 85.71 only occurring for pods that contained 6 or fewer total seeds (**Table 5**).

**Table 5:** Number and percentage of *Brassica napus* seeds automatically sorted into the correct pod valve.

Seed Pod ID	Valve 1		Valve 2		Number of Correctly Sorted Seeds	Percentage of Correctly Sorted Seeds (%)
	True Number of Seeds	Predicted Number of Seeds	True Number of Seeds	Predicted Number of Seeds		
**C0007186**	4	5	1	0	4	80
**C0007197**	9	10	10	10	19	100
**C0007198**	3	3	1	1	4	100
**C0007205**	1	2	1	0	1	50
**C0007224**	16	16	18	18	34	100
**C0007226**	3	3	3	3	6	100
**C0007239**	2	3	6	5	7	87.5
**C0007256**	17	17	13	13	30	100
**C0007262**	2	2	0	0	2	100
**C0007269**	0	0	1	1	1	100
**C0007274**	18	19	16	15	33	97.06
**C0007299**	0	0	3	3	3	100
**C0007311**	8	8	7	7	15	100
**C0007420**	5	4	2	3	6	85.71
**C0007440**	11	12	10	7	18	85.71
**C0007456**	3	2	1	2	3	75
**C0007853**	4	3	2	3	5	83.33
**C0007864**	1	1	3	3	4	100
**C0007865**	1	2	3	2	3	75
**Total**	**108**	**112**	**101**	**96**	**198**	**94.74**

## 4 Discussion

The overall accuracy of the StarDist-3D model fine-tuned on 3D µCT images of *B. napus* pods was higher than reported for the developmental use case of detection and segmentation of individual cell nuclei (Weigert et al., 2020). This may be due to the very high resolution of 3D µCT images, and smaller number of target objects within them, which meant that individual seeds, as opposed to cell nuclei, were less likely to be obscured, and there were few cases of closely clustered target objects. The high precision of the fine-tuned StarDist-3D model may also be due to the fact that no other pod structures within the images closely resemble seeds in shape. This demonstrates the suitability of the 3D µCT *B. napus* pod image dataset to automated data extraction. A small number of false negative errors occurred when seeds were much smaller and unevenly shaped compared to the majority of seeds, possibly due to post-fertilization seed abortion. Dissection of the pods to determine the cause of this size and shape disparity in these missed seeds, and further fine-tuning of the pre-trained StarDist-3D model based on a curated dataset of seeds of more diverse size and shape is likely to improve the recall rate. However, the low error rate already achieved with a relatively small amount of annotated training data suggests the high resolution of the 3D µCT *B. napus* pod images make them a valuable resource highly compatible with state-of-art computer vision approaches.

The fine-tuned StarDist-3D approach utilized in this paper allowed for accurate data on the number, spatial arrangement, size and shape of seeds to be extracted from a 3D µCT image of a whole *B. napus* pod in under 1 minute 30 seconds, as opposed to manual image analysis methods which took approximately 14 minutes to obtain only a small subset of the measurements. Manual methods are more labour intensive as pods have to be collected and placed flat with a contrasting background to obtain high-quality images for semi-automatically measuring valve and beak length by using SmartRoot tool in Fiji (Schindelin et al., 2012). Then, the pods have to be manually opened to obtain SNPP data. The manual process scan can result in loss of seeds and inaccurate counting. Opening the pod invariably leads to movement of the seed within the pod, therefore information on the valve and spacing is lost. Hence, the automated pipeline that we describe here is more efficient and less time consuming than currently used methods. In addition, the automatic seed sorting by valve step also allowed for data to be collected on the spatial arrangement of seeds in relation to both each other and pod valves that cannot be examined through conventional dissection methods of examining pods. For pods with a very low number of seeds (six or less) valve misclassification errors were more common, demonstrating that it was difficult to reliably predict the position and shape of pseudoseptum from a small number of data points on seed location. Future development of an additional edge detection algorithm to directly detect the pseudoseptum, rather than relying solely on seed position, could improve the accuracy of valve sorting for pods with very few seeds. However, this study demonstrates the significantly less computationally intensive graphical method of predicting pseudoseptum shape and seed valve position using lowess regression is suitable for analysis of images of pods with 7 seeds or more.

The reduction in the bottleneck for analysing the 3D µCT image dataset provided by the StarDist-3D approach could enable detailed data on the number, size, shape and spatial arrangement of seeds to be integrated into models of plant development. It could also potentially be applied to 3D µCT imagery of seeds from other species or other plant structures with relatively little retraining effort provided the target objects can appropriately be described as star-convex polygons. The gradient of seed growth within a pod, the difference of seed growth and abortion within pods in different positions in the main inflorescence, comparison of pods between the main and the secondary inflorescences and the effect of different environmental perturbations, such as heat stress, could be studied. Hence, this method of analysis that does not require opening pods will help to better understand SNPP and seed abortion and their relation to plant seed yield in several crops. A multi-class version of the StarDist-3D model could be trained to predict shape and position of beaks, pedicels and post-fertilisation aborted seeds as these structures can also be accurately reconstructed as star-convex polygons. This will allow the automatically extraction of further metrics such as beak length and overall pod length, as well as reduce the potential of false negative errors caused by aborted seeds that tend to be unusually small and irregularly shaped compared to mature seeds. This extension would rely upon annotating a larger number of full 3D µCT as each only contains a single beak and pedicel, however, since accurate results were obtained with the number of seeds labelled for training data in this study researchers may only need to label beaks and pedicels in further imagery in order to prepare an adequate multi-class training dataset.

Scale has been shown to have negligible impact on accuracy of StarDist-3D object detection and segmentation, therefore the fine-tuned model described could reliably be applied to images of B. napus seed pods that vary significantly in size, both in terms of the pods themselves and size of individual seeds. The findings of this study also demonstrated that the Stardist-3D method could be applied to imagery of seed from other plant species, as well as other plant structures such as peas, nuts or grains that are rounded or ovate in shape and therefore can be accurately reconstructed as star-convex polyhedra. This potentially includes more unusually shaped, less spherical seeds and nuts from species such as *Arabidopsis thaliana*, *Camelina sativa* and *Arachis hypogaea* (peanuts) as the anisotropy, or non-spherical irregularities of these seeds, could be taken into account with the Stardist-3D method.

The fine-tuned StarDist-3D model can also likely be reliably applied to datasets with lower contrast than the Scanco µCT images used in this study, as the StarDist-3D methods have been demonstrated to yield more accurate results for low contrast, low signal-to-noise 3D volume data compared to other contemporary deep learning based instance segmentation methods (Schmidt et al., 2018; Weigert et al., 2020). It is recommended that data augmentation methods be applied to the study dataset in future to explore the effect of resolution on performance, as at a sufficiently low resolution the accuracy of shape prediction may be impacted due to the borders of seeds being blurred (Schmidt et al., 2018; Weigert et al., 2020).

The automated clustering of the segmented images of individual seeds that are output by the fine-tuned StarDist-3D with a rotationally invariant method can also be explored, as a high degree of variation was observed in the internal structure of the seeds. The segmented seed images would need to be represented as rotationally invariant images in order to explore clustering, as seeds are oriented at different directions within pods and rotationally invariant representation would negate the effect of these differing orientations so that other similarities and differences in the internal appearance of the seeds could be quantified (Zhao & Singer 2014). This is a very promising step once images with higher resolution are acquired. Clustering could reveal similarities in internal appearance between groups of seeds that could be linked to biological origins, which could be ground-truthed through manual examination and seed dissection after 3D µCT. Moreover, synchrony and different orientation of the seeds could be further explored. This knowledge is of high importance as breeders pursue good and synchrony of seed maturation. Therefore, links between these traits and the growing conditions and genotypes of the plants that the seeds were collected from can be explored in order to better understand factors affecting seed maturation and plant yield.

## 5 Conclusion

High-throughput plant image datasets have the potential to greatly improve our understanding of the factors affecting dynamic responses in plant development and crop yield, however a lack of reliable and efficient methods for extracting phenotype data from these datasets remains a major bottleneck. This paper demonstrates that an existing, state-of-the-art object detection and segmentation method, StarDist-3D, can be applied with little modification to automatically obtain seed number, size, shape and spatial seed arrangement from 3D µCT images of *B. napus* pods in a time-saving and non-destructive manner with a high degree of accuracy. This method could enable the study of seed development within a specific time-point or during different phases of pod growth, obtaining very specific and detailed information that otherwise, would not be possible to accurately capture. Acquiring information regarding the internal structure of opaque pods can be incorporated into high-throughput plant phenotyping platforms and enables the opportunity of understanding and linking pod, seed development and disposition within different germplasm and plant developmental responses to biotic and abiotic stresses. The findings of this paper also demonstrate how current gold standard computer vision methods can be generalised to accurately analyse imagery collected using a variety of sensors, at different scales and from a wide range of scientific domains.

## Data availability statement

The raw data supporting the conclusions of this article will be made available by the authors, without undue reservation.

## Author contributions

EC annotated training data, led development of the automated plant phenotyping pipeline, analyzed results of the pipeline, and wrote the first draft. LS and SK collected the plant image datasets. LS, SK, and SA contributed to scoping the developing the automated plant phenotyping pipeline and reviewing and editing the manuscript. All authors contributed to the article and approved the submitted version.
