# Supplementary figures and images for: Automated extraction of pod phenotype data from micro-computed tomography
Source: Front Plant Sci. 2023 Feb 24;14:1120182. doi: 10.3389/fpls.2023.1120182 (PMC9998914; doi:10.3389/fpls.2023.1120182)

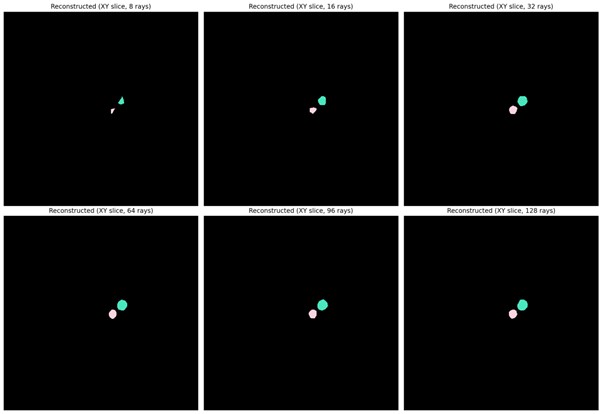

Supplement: Supplementary file 1 [file Image_1.jpeg]
